# Characteristics of Patients with Unrecognized Sleep Apnea Requiring Postoperative Oxygen Therapy

**DOI:** 10.3390/jpm12101543

**Published:** 2022-09-20

**Authors:** Edwin Seet, Rida Waseem, Matthew T. V. Chan, Chew Yin Wang, Vanessa Liao, Colin Suen, Frances Chung

**Affiliations:** 1 Department of Anaesthesia, Yong Loo Lin School of Medicine, National University of Singapore, Singapore 117559, Singapore; 2 Department of Anaesthesia, Khoo Teck Puat Hospital, National Healthcare Group, Singapore 768828, Singapore; 3 Lee Kong Chian School of Medicine, Nanyang Technological University, Singapore 637718, Singapore; 4 Department of Anesthesiology and Pain Medicine, Toronto Western Hospital, University Health Network, University of Toronto, Toronto, ON M5T 1R8, Canada; 5 Department of Anaesthesia and Intensive Care, Chinese University of Hong Kong, Hong Kong SAR 999077, China; 6 Department of Anaesthesiology, Faculty of Medicine, University of Malaya, Kuala Lumpur 50603, Malaysia; 7 University of Western Ontario, London, ON N6A 3K7, Canada; 8 University of Toronto, Toronto, ON M5S 3E5, Canada

**Keywords:** oxygen therapy, obstructive sleep apnea, personalized medicine, hypoxemia, postoperative care, oximetry parameters, phenotypes

## Abstract

Surgical patients with obstructive sleep apnea (OSA) have increased risk of perioperative complications. The primary objective is to determine the characteristics of surgical patients with unrecognized OSA requiring oxygen therapy for postoperative hypoxemia. The secondary objective is to investigate the characteristics of patients who were responsive to oxygen therapy. This was a post-hoc multicenter study involving patients with cardiovascular risk factors undergoing major non-cardiac surgery. Patients ≥45 years old underwent Type 3 sleep apnea testing and nocturnal oximetry preoperatively. Responders to oxygen therapy were defined as individuals with ≥50% reduction in oxygen desaturation index (ODI) on postoperative night 1 versus preoperative ODI. In total, 624 out of 823 patients with unrecognized OSA required oxygen therapy. These were mostly males, had larger neck circumferences, higher Revised Cardiac Risk Indices, higher STOP-Bang scores, and higher ASA physical status, undergoing intraperitoneal or vascular surgery. Multivariable regression analysis showed that the preoperative longer cumulative time SpO_2_ < 90% or CT90% (adjusted *p* = 0.03), and lower average overnight SpO_2_ (adjusted *p* < 0.001), were independently associated with patients requiring oxygen therapy. Seventy percent of patients were responders to oxygen therapy with ≥50% ODI reduction. Preoperative ODI (19.0 ± 12.9 vs. 14.1 ± 11.4 events/h, *p* < 0.001), CT90% (42.3 ± 66.2 vs. 31.1 ± 57.0 min, *p* = 0.038), and CT80% (7.1 ± 22.6 vs. 3.6 ± 8.7 min, *p* = 0.007) were significantly higher in the responder than the non-responder. Patients with unrecognized OSA requiring postoperative oxygen therapy were males with larger neck circumferences and higher STOP-Bang scores. Those responding to oxygen therapy were likely to have severe OSA and worse preoperative nocturnal hypoxemia. Preoperative overnight oximetry parameters may help in stratifying patients.

## 1. Introduction

Postoperative patients are susceptible to hypoxemia because of incomplete lung re-expansion, reduced chest wall and diaphragmatic activity caused by the surgical insult and pain, residual effects of anesthetic drugs, and use of opioids. This may result in ventilation–perfusion mismatch, alveolar hypoventilation, and upper airway obstruction [1].

Obstructive sleep apnea (OSA) is a common sleep-disordered breathing characterized by repetitive pharyngeal collapse. The prevalence of unrecognized OSA in patients undergoing elective surgery is estimated to be at least 50% [2]. Surgical patients with OSA are prone to increased postoperative complications [3,4,5,6,7,8]. In an international prospective cohort study of patients undergoing major non-cardiac surgery, patients with unrecognized OSA had fifty percent increased risk of postoperative cardiovascular events [2,4]. The mean cumulative duration of oxyhemoglobin desaturation less than 80% during the postoperative nights in patients with cardiovascular complications was longer than in those without. Overnight or nocturnal oximetry is a valid tool in screening surgical patients for OSA using the oxygen desaturation index (ODI) [9,10,11]. Furthermore, hypoxemia detected by oximetry has been shown to predict postoperative cardiovascular events in surgical patients with OSA [12].

Continuous positive airway pressure (CPAP) is a standard treatment for patients with OSA. Auto-titrated CPAP therapy decreases the postoperative apnea hypopnea index (AHI) and improves oxygenation in patients with moderate to severe OSA [13]. However, CPAP is often not tolerated by patients with a low adherence rate of 50–60% [13,14,15,16]. In clinical practice, surgical patients with OSA who developed postoperative hypoxemia often require supplemental oxygen therapy postoperatively in the wards. It is used as an alternative therapy for those patients who are non-adherent to CPAP, newly diagnosed patients without adequate time to initiate CPAP therapy, or patients with suspected OSA. A recent randomized controlled trial involving patients with newly diagnosed OSA showed that postoperative oxygen therapy improved oxygenation and decreased the AHI [17].

The concept of precision medicine and personalized OSA therapy has emerged, based on the premise that OSA interventions have maximal impact when they match patients’ underlying phenotypes [18]. Sands et al. found that patients with OSA may benefit from stabilizing ventilatory control with supplemental oxygen therapy [19]. Patients were classified as “responders” to oxygen if their AHI was reduced by ≥50% with supplementary oxygen therapy (a priori criterion).

To date, we do not know the characteristics of surgical patients with unrecognized or newly diagnosed OSA who may benefit from postoperative oxygen therapy. The objective of the study is to elucidate the phenotypes of surgical patients with unrecognized OSA who developed postoperative hypoxemia on postoperative night 1 (N1). The secondary aim is to investigate the characteristics of surgical patients with unrecognized OSA who respond to oxygen therapy.

## 2. Methods

This was a planned, post hoc analysis of the multicenter prospective cohort Postoperative vascular complications in unrecognized Obstructive Sleep Apnea (POSA) study of patients undergoing major non-cardiac surgery [2,4]. The study was conducted in five countries at eight hospitals from January 2012 to July 2017. Ethics approval was obtained by all participating institutions. All patients gave informed consent. The study met the Declaration of Helsinki guidelines. Details of the methods were previously published [2].

Patients undergoing major elective non-cardiac surgery were approached for recruitment. The inclusion criteria were (1) age ≥45 years undergoing major noncardiac surgery (intraperitoneal, major orthopedic, or vascular); and (2) at least one risk factors for postoperative cardiovascular events (i.e., history of coronary artery disease, heart failure, stroke or transient ischemic attack, diabetes requiring treatment, and renal impairment with a preoperative plasma creatinine concentration >175 μmol/L). The exclusion criteria were (1) prior diagnosis or undergoing corrective surgery for OSA; and (2) patients requiring greater than two days of mechanical lung ventilation post-surgery.

### 2.1. Home Sleep Apnea Testing and Pulse Oximetry

All patients in this study underwent a preoperative overnight sleep study at home or in the hospital using type 3 home sleep apnea testing (ApneaLink Plus; ResMed, San Diego, CA, USA). It includes a nasal pressure transducer that measures flow limitation and snoring, and records on a 16-bit signal processor at a sampling rate of 100 Hz. In addition to the home sleep apnea testing, oxyhemoglobin saturation (SpO_2_) was simultaneously recorded using a high-resolution pulse oximetry wristwatch (PULSOX-300i, Konica Minolta Sensing, Inc., Osaka, Japan).

The data were extracted the next morning using ApneaLink and Profox (Profox Associates, Escondido, CA, USA) software, respectively. The data were processed by a technician blinded to the clinical data and the STOP-Bang score [4]. The sleep parameters were extracted from ApneaLink Plus. The sleep-associated apnea and hypopnea events were scored according to the American Academy of Sleep Medicine criteria [20]. Apnea was defined as an airflow reduction of ≥90% for ≥10 s from the baseline. Hypopnea was defined as a reduction in airflow for ≥30% for ≥10 s from the baseline and associated with ≥3% oxyhemoglobin desaturation. Patients with an AHI ≥5 events/h were considered to have OSA.

The ODI, cumulated time of SpO_2_ < 90% (CT90%), cumulated time of SpO_2_ < 80% (CT80%), lowest SpO_2_, and average overnight SpO_2_ were extracted from the oximetry using Profox software. ODI is defined as the number of events per hour with at least a 4% decrease in saturation from the average saturation in the preceding 120 s for at least 10 s. The oximetry recording data were processed, which were recorded between 00:00 a.m. and 06:00 a.m. at night, although it was not known if the patients were asleep during this entire period [9]. Oximetry-derived parameters of preoperative and postoperative N1 were used for the analysis.

### 2.2. Procedures

All types of anesthetic techniques were permitted, and surgery was performed according to the routine standard of care at each site. Following surgery, supplemental oxygen was administered by various devices in the surgical wards. The types of devices include a nasal cannula, simple facemask, non-breathing mask, and non-invasive ventilation devices, the latter including continuous positive airway pressure (CPAP) and bilevel positive airway pressure (BPAP) ventilation. The types of devices used, and the duration of oxygen therapy, were collected. In the surgical wards, the clinical decision on whether supplemental oxygen therapy was given, and the type of devices used, was determined by a healthcare team as per local standard practice. Among the participated hospitals, the threshold criteria for the administration of supplemental oxygen therapy ranged from SpO_2_ ≤ 92% to SpO_2_ ≤ 94% in the surgical wards. The healthcare team was blinded to the results of the STOP-Bang score, preoperative home sleep apnea testing, or oximetry.

### 2.3. Data Collection

Prior to surgery, the demographic characteristics, neck circumference, comorbidities, American Society of Anesthesiologists (ASA) physical status, and Revised Cardiac Risk Indices were collected [2,4]. The patients’ risk for OSA were assessed using the STOP-Bang (Snoring, Tiredness, Observed Apnea, High Blood Pressure, Body Mass Index, Age, Neck Circumference, and Gender) screening tool (scores range from 0 to 8, with a score of 0–2 indicating low risk, 3–4 intermediate risk, and 5–8 high risk) [21]. The types of surgery and anesthesia were collected. Patients were classified as responders and non-responders to supplemental oxygen therapy. Responders to oxygen therapy were defined as individuals with ≥50% reduction (a priori criterion) in postoperative ODI versus preoperative ODI, while non-responders were defined as <50% reduction in ODI on postoperative N1 [19].

### 2.4. Statistical Analysis

The analyses were conducted using STATA (v.14.2). Demographic and oximetry variables were presented using descriptive statistics. Continuous variables were reported using means and standard deviations, while categorical variables were presented using frequencies and percentages. Independent sample *t*-test or Chi-square test were used to examine the differences between the characteristics of patients as appropriate. Multivariable logistic regression was conducted to identify the characteristics of patients requiring oxygen therapy. Covariates were added in the model if they were significant in the univariate analysis. A *p*-value < 0.05 was considered statistically significant. The sample size of the study was based on the primary outcome of the original study [2,4].

## 3. Results

### 3.1. Patient Characteristics

**Figure 1:**
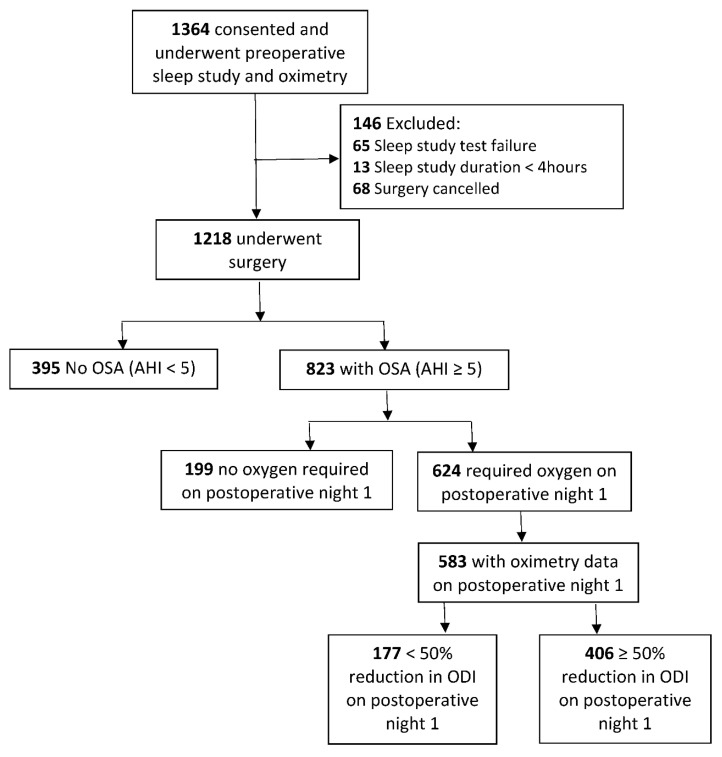
Study flow chart. Abbreviations: OSA, obstructive sleep apnea; AHI, apnea hypopnea index; ODI, oxygen desaturation index.

Overall, 1364 patients were recruited for the original study, with 1218 patients that underwent surgery. Data from 395 patients with no OSA were excluded, as it does not pertain to our current research question. A total of 823 patients with unrecognized OSA (AHI ≥ 5 events per hour) were included for analyses (Figure 1).

**Figure 2:**
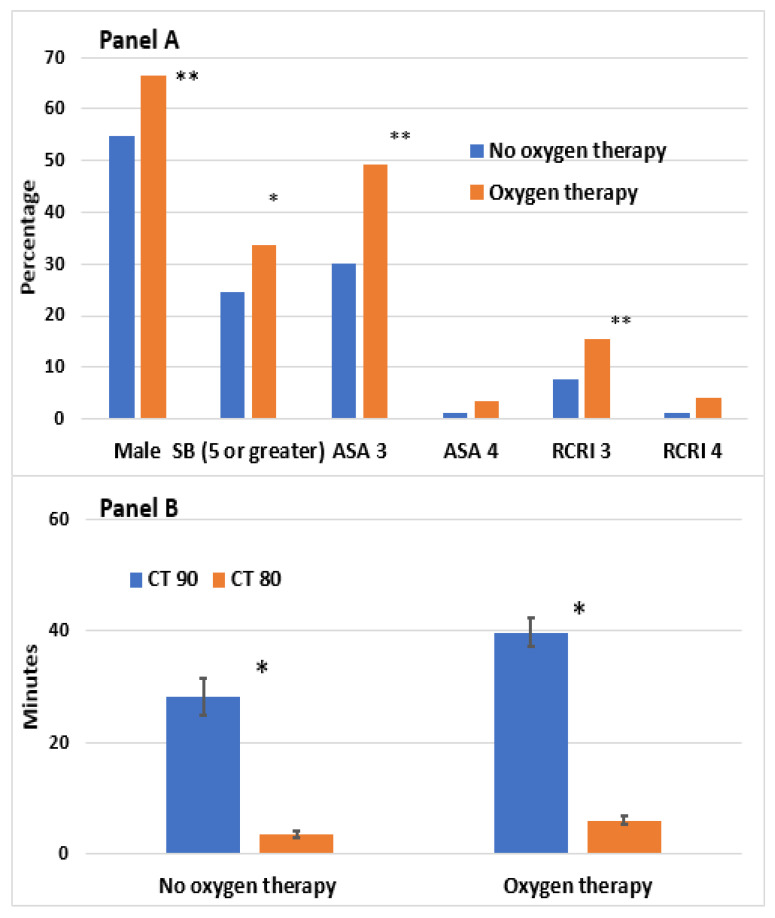
Characteristics of OSA patients receiving oxygen. Vertical bar represents the standard error of the mean. Panel (**A**) shows the demographic characteristics of patients receiving oxygen therapy. Panel (**B**) shows the oximetry parameters of patients receiving oxygen therapy. Abbreviations: SB: STOP-Bang score; RCRI: revised cardiac risk index; CT: cumulative time; SpO_2_: average oxyhemoglobin saturation; ODI: oxygen desaturation index; OSA, obstructive sleep apnea. * *p* < 0.05, ** *p* < 0.01.

The baseline demographics, comorbidities, and oximetry parameters for 823 patients with unrecognized OSA are described in Table 1. Of the 823 patients, 624 (76%) required oxygen therapy and 199 (24%) were not on oxygen therapy during postoperative N1. The mean age and body mass index (BMI) of patients were 67.8 ± 9.2 years and 27.1 ± 5.3 kg/m^2^ with 63.7% male. The mean ODI was 17.3 ± 12.5 events/h, while the lowest SpO_2_, CT90%, and CT80% were 75.1 ± 11.2%, 36.9 ± 59.6 min, and 5.4 ± 17.2 min, respectively (Table 1) (Figure 2).

In total, 583 out of 624 patients (93.9%) had oximetry data on postoperative N1 available for analysis of responsiveness to oxygen therapy (Figure 1); 66.7% of patients were administered nasal cannula, 24.4% simple face mask, 8.4% non-invasive ventilation devices, and less than 1% non-breathing mask (Supplementary Table S1). On N1, the mean duration of oxygen therapy for 583 patients was 12.5 ± 5.6 h. Eleven percent of patients with moderate OSA or severe OSA required non-invasive ventilation devices postoperatively while 6% of patients with mild OSA required non-invasive ventilation devices (Supplementary Table S1).

### 3.2. Characteristics of Unrecognized OSA Patients Requiring Postoperative Oxygen Therapy

On postoperative N1, patients with unrecognized OSA who required oxygen therapy were associated with male gender, larger neck circumferences, greater STOP-Bang scores, higher ASA physical status, and higher revised cardiac risk indices. Procedures associated with oxygen therapy were higher risk and longer duration operations, such as intraperitoneal or vascular surgeries under general anesthesia (Table 1). There was no difference in preoperative AHI or ODI in those who required oxygen therapy compared to those who did not. The preoperative overnight average SpO_2_ was significantly lower (93.9 ± 3.0 vs. 94.9 ± 1.8%, *p* < 0.001), and CT90% (39.7 ± 63.4 vs. 28.2 ± 44.7 min, *p* < 0.005) and CT80% (6.0 ± 19.0 vs. 3.5 ± 9.1 min, *p* < 0.013) were significantly longer in those requiring oxygen therapy versus those who did not (Table 1).

Multivariable logistic regression analysis showed that the ASA 3 physical status was associated with 80% higher risk in patients with OSA requiring oxygen therapy (adjusted odds ratio (aOR) 1.77, 95% confidence interval (CI): 1.21–2.56, *p* < 0.003) (Table 2). Similarly, higher revised cardiac risk indices of 2, 3, and 4/5 were associated with OSA patients requiring oxygen therapy (aOR 2.81, 95% CI: 1.91–4.13, *p* < 0.001; aOR 3.52, 95% CI:1.87–6.61, *p* < 0.001; and aOR 6.04, 95% CI: 1.27–28.7, *p* < 0.024, respectively) (Table 2). For the preoperative oximetry parameters, a lower preoperative average overnight SpO_2_ (aOR 0.75, 95% CI: 0.67–0.84, *p* < 0.001) and a longer CT90% (aOR 0.96, 95% CI: 0.99–1.00, *p* = 0.030) were associated with OSA patients requiring oxygen therapy (Table 2).

### 3.3. Identification of Responders to Oxygen Therapy

On postoperative N1, 70% (406/583) of patients with unrecognized OSA were responders to oxygen therapy, with a ≥ 50% reduction in postoperative ODI versus preoperative ODI, while 30% (177/583) were non-responders, with a <50% ODI reduction. There was no significant difference in demographic or anthropometric difference by age, gender, BMI, and neck circumference between the responders and non-responders. Responders to oxygen therapy had higher STOP-Bang scores and revised cardiac risk indices (Table 3). A higher proportion of patients in the responder group underwent longer duration intraperitoneal surgery (Table 3).

Preoperative AHI (19.5 ± 14.4 vs. 14.4 ± 12.1 events per hour, *p* < 0.001), ODI (19.0 ± 12.9 vs. 14.1 ± 11.4 events per hour, *p* < 0.001), CT90% (42.3 ± 66.2 min vs. 31.1 ± 57.0 min, *p* = 0.038), and CT80% (7.1 ± 22.6 vs. 3.6 ± 8.7 min, *p* = 0.007) were significantly higher among responders vs. non-responders, indicating that the severity of unrecognized OSA was greater and the degree of preoperative hypoxemia was worse in the responders (Table 3). Correspondingly, the postoperative ODI was significantly lower in the responders than the non-responders (2.9 ± 3.1 events per hour vs. 15.4 ± 12.2 events per hour, *p* < 0.001).

## 4. Discussion

In this planned post hoc study, we determined the characteristics of patients with unrecognized OSA requiring postoperative oxygen therapy. Surgical patients with unrecognized OSA who had higher revised cardiac risk indices and higher ASA physical status scores were independently associated with the need for supplemental oxygen therapy in postoperative N1. Preoperative overnight oximetry values, such as a lower average overnight SpO_2_ and longer CT90%, were significantly associated with OSA patients requiring oxygen therapy, whereas OSA severity indicators, such as AHI and ODI, were not.

Furthermore, we investigated the subgroup of patients with good clinical response to supplemental oxygen. Seventy percent of patients were responders, and these had higher STOP-Bang scores, higher revised cardiac risk indices, and undergoing longer-duration intraperitoneal surgery than non-responders. They were more likely to have severe OSA and worse preoperative hypoxemia, indicated by higher preoperative ODI, CT90%, and CT80%. Knowledge of the characteristics of patients with unrecognized OSA who required postoperative oxygen therapy and the traits of responders will help in clinical decision making and perioperative risk mitigation.

Preoperative overnight or nocturnal oximetry is a validated screening tool for individuals at risk of OSA undergoing major non-cardiac surgery with cardiovascular risk factors [11]. Oximetry had good predictive validity in screening OSA for surgical patients at risk of cardiovascular events [11]. ODI ≥ 15 events per hour has a sensitivity and specificity of 88.4% and 95.4% to identify moderate to severe OSA. We recently showed that oximetry parameters, including preoperative ODI ≥ 30 events/h and CT80% ≥ 10 min, are associated with an increased risk of postoperative cardiovascular events [12].

In our study on surgical patients, response to oxygen therapy was not correlated with demographic and anthropometric characteristics such as age, gender, BMI, and neck circumference. These results are consistent with a recent study identifying traits associated with responsiveness to oxygen therapy in diagnosed sleep apnea patients [19]. Importantly, we found that preoperative AHI from sleep apnea testing, and baseline ODI, CT90%, and CT80% from preoperative overnight oximetry, were significantly higher in the responder group. Thus, the responders were likely to have severe OSA and worse preoperative hypoxemia. These oximetry-derived parameters can help determine patients who would require postoperative supplemental oxygen and have good response to oxygen therapy clinically.

A significant challenge in the management of OSA patients is low adherence to standard treatment such as CPAP. Perioperative CPAP adherence is approximately 50–60% [6,13,14,15]. Based on the profile of patients elucidated in this study, postoperative supplemental oxygen could potentially be used for those who are CPAP non-adherent, newly diagnosed patients, or suspected OSA. Most of the sequelae of OSA are more strongly linked to the degree and duration of oxygen desaturation than to the number of apneas and hypopneas or disruptions in sleep architecture [22]. In a prospective trial of 123 surgical patients randomized to oxygen or no oxygen therapy, postoperative supplemental oxygen was found to improve oxygenation and decrease AHI without increasing the duration of an apnea–hypopnea event [17]. Notably, a small number (11.4%) of patients experienced substantial carbon dioxide retention. Supplemental oxygen also improved sleep-related disturbances and oxygenation in patients with OSA living in high-altitude regions [23]. Patients who failed to respond to upper airway surgery for OSA can benefit from oxygen therapy [24]. In two systematic reviews and meta-analyses, oxygen therapy significantly improved oxygen saturation in medical patients with OSA [25,26]. One concern for surgical patients with OSA is that supplemental oxygen could lead to longer apnea events with associated hypercapnia and sustained hypoventilation [25]. Hypercapnia may be worsened by supplemental oxygen in patients with obesity hypoventilation syndrome (OHS), which is present in 10% to 20% of patients with OSA [27,28].

Supplemental oxygen was shown to decrease AHI in patients with ventilatory instability (high loop gain), but not in patients with a low loop gain [29]. Sands et al. demonstrated that besides ventilatory instability, reduced upper airway collapsibility and compensation were associated with oxygen responsiveness in OSA patients [19]. These phenotypic traits could potentially form the basis for a personalized approach to postoperative oxygen therapy for patients with OSA [18]. Further work is needed to determine whether patients with OSA caused by other pathophysiologic processes would benefit from supplemental oxygen.

Ventilatory chemosensitivity may be a predictor of opioid-induced respiratory depression [30], and increased inspired oxygen may result in greater opioid-induced respiratory depression [31]. When supplemental oxygen is given, it may mask the ability of oximetry to detect abnormalities in ventilation [31,32]. Oxygen therapy may therefore be a double-edged sword: on one hand, it may improve oxygen saturation, but on the other hand, it can potentially increase the risk of hypercarbia [17]. Additional methods for detecting hypoventilation, such as continuous measurement of respiratory rate and end-tidal carbon dioxide monitoring, may be needed. Nevertheless, supplemental oxygen is a useful therapy for postoperative hypoxemia and to maintain overnight oxygenation in surgical patients with OSA [17,25,26].

## 5. Limitations

There are some limitations in this study. As portable sleep apnea testing was used to determine the preoperative sleep parameters, we were unable to determine the arousal index and other parameters, which would give precise insight into the phenotypes of surgical patients with unrecognized OSA in their response to oxygen therapy. Portable sleep apnea testing and overnight oximetry did not monitor electroencephalography, and we did not know whether patients were asleep when data were collected. Wakefulness during the sleep apnea testing and overnight oximetry may result in underestimation of AHI and ODI, and the severity of sleep apnea. Patients’ response to oxygen therapy is a new field in personalized medicine and our post-hoc analyses may be construed as hypothesis generating.

## 6. Conclusions

Patients with unrecognized OSA requiring oxygen therapy were mostly males, had larger neck circumferences, and higher STOP-Bang scores, as well as underwent longer-duration intraperitoneal or vascular surgery. CT90% and lower average overnight SpO_2_ were independently associated with patients requiring postoperative oxygen therapy. Seventy percent of patients with unrecognized OSA were responders to oxygen therapy. The responders were likely to have severe OSA and worse preoperative hypoxemia, indicated by higher preoperative ODI, CT90%, and CT80%. Therefore, preoperative oximetry-derived parameters are useful in stratifying patients—to determine which patients would likely require postoperative supplemental oxygen, as well as which patients would have a good clinical response to oxygen therapy. This approach can be readily implemented in the clinical setting to personalize perioperative patient care.

## Figures and Tables

**Table 1:** Characteristics of OSA patients requiring oxygen therapy versus no oxygen therapy on postoperative night 1.

Characteristics	Total (n) 823	No Oxygen Therapy (n) 199	Oxygen Therapy (n) 624	*p* Value
**Age, years**	67.8 ± 9.2	67.0 ± 9.4	68.1 ± 9.2	0.111
**Gender, male**	524 (63.7)	109 (54.8)	415 (66.5)	*0.003*
**BMI, kg/m^2^**	27.1 ± 5.3	27.2 ± 5.4	27.1 ± 5.3	0.887
**Neck circumference, cm**	39.1 ± 3.4	38.2 ± 3.4	39.4 ± 3.4	*<0.001*
**STOP-Bang score**				*0.015*
**Low risk (0–2)**	132 (16.0)	42(21.1)	90 (14.4)	
**Intermediate (3–4)**	431 (52.4)	108 (54.3)	323 (51.8)	
**High (5–8)**	260 (31.6)	49 (24.6)	211 (33.8)	
**ASA**				*<0.001*
**2**	432 (52.5)	137(68.8)	295 (47.3)	
**3**	367 (44.6)	60 (30.2)	307 (49.2)	
**4**	24 (2.9)	2 (1)	22 (3.5)	
**Revised Cardiac Risk** **Index**				*<0.001*
**1**	343 (41.7)	121 (60.8)	222 (35.6)	
**2**	342 (41.6)	61 (30.7)	281 (45.0)	
**3**	111 (13.5)	15 (7.5)	96 (15.4)	
**4/5**	27 (3.3)	2 (1.0)	25 (4.0)	
**Type of Surgery**				*<0.001*
**Intraperitoneal** **Vascular ** **Orthopedic ** **Other**	261 (31.7) 125 (15.2) 263 (32.0) 174 (21.1)	46 (23.1) 18 (9.0) 86 (43.2) 49 (24.6)	215 (34.5) 107 (17.1) 177 (28.4) 125 (20.0)	
**Duration of surgery, hours**	2.89 ± 2.06	2.10 ± 1.40	3.14 ± 2.17	*<0.001*
**Duration of anesthesia, hours**	3.78 ± 2.28	2.84 ± 1.65	4.10 ± 2.38	*<0.001*
**Type of anesthesia**				*<0.001*
**GA** **Combined** **Neuraxial**	559 (67.9) 78 (9.5) 186 (22.6)	109 (54.8) 14 (7.0) 76 (38.2)	450 (72.1) 64 (10.3) 110 (17.6)	
	**Preoperative sleep study parameters**			
**AHI** **≥** **5–<15**	452 (54.9)	116 (58.3)	336 (41.0)	0.251
**AHI** **≥15–<30**	228 (27.7)	46 (23.1)	182 (29.2)	
**AHI** **≥ 30**	143 (17.4)	37 (18.6)	106 (17.0)	
**AHI, events/hr**	18.0 ± 14.1	17.6 ± 14.1	18.1 ± 14.0	0.651
	**Preoperative overnight oximetry parameters**			
**ODI preop, events/hour**	17.3 ± 12.5	16.4 ± 12.3	17.5 ± 12.5	0.260
**Average SpO_2_, %**	94.2 ± 2.8	94.9 ± 1.8	93.9 ± 3.0	*<0.001*
**Lowest SpO_2_, %**	75.1 ± 11.2	75.3 ± 11.0	75.1 ± 11.3	0.831
**CT 90%, minutes**	36.9 ± 59.6	28.2 ± 44.7	39.7 ± 63.4	*0.005*
**CT 80%, minutes**	5.4 ± 17.2	3.5 ± 9.1	6.0 ± 19.0	*0.013*
**Postoperative ODI N1, events/hour**	8.6 ± 10.9	14.6 ± 13.4	6.7 ± 9.2	*<0.001*

Independent sample *t*-test used to compare numerical values and Chi-square test used to compare categorical values. CT: cumulative time; SpO_2_: oxyhemoglobin; ODI: oxygen desaturation index; AHI: apnea–hypopnea index; ICU: intensive care unit; BMI: body mass index; OSA: obstructive sleep apnea; ASA: American Society of Anesthesiologists Physical Status.

**Table 2:** Multivariable logistic regression analysis for patients with unrecognized OSA requiring oxygen therapy.

Variables	Unadjusted		Adjusted	
	Odds Ratio (95% CI)	*p* Value	Odds Ratio (95% CI)	*p* Value
**ASA**				
**2**	1 [reference]			
**3**	2.38 (1.69–3.35)	<0.001	1.77 (1.21–2.56)	*0.003*
**4**	5.11 (1.18–22.0)	0.029	2.08 (0.45–9.68)	0.350
**Revised Cardiac Risk Index**				
**1**	1 [reference]			
**2**	2.51 (1.76–3.58)	<0.001	2.81 (1.91–4.13)	*<0.001*
**3**	3.49 (1.94–6.28)	<0.001	3.52 (1.87–6.61)	*<0.001*
**4/5**	6.81 (1.59–29.3)	0.010	6.04 (1.27–28.7)	*0.024*
**STOP-Bang score**				
**Low (0–2)**	1 [reference]			
**Intermediate (3–4)**	1.40 (0.91–2.14)	0.125	1.22 (0.77–1.94)	0.393
**High (5–8)**	2.01 (1.24–3.25)	0.004	1.62 (0.96–2.74)	0.072
**Preop average overnight SpO_2_, %**	0.82 (0.75–0.89)	<0.001	0.75 (0.67–0.84)	*<0.001*
**Preop CT90, min**	1.00 (1.00–1.01)	0.020	0.96 (0.99–1.00)	*0.030*

CT: cumulative time; SpO_2_: oxyhemoglobin; CI: confidence interval; ASA: American Society of Anesthesiologists Physical Status.

**Table 3:** The clinical characteristics of patients with unrecognized OSA based on responsiveness to postoperative oxygen therapy.

Characteristics N = 583	Non-Responders 177	Responders 406	*P*
**Age, years**	68.0 ± 8.7	68.4 ± 9.2	0.567
**Gender, male**	118 (66.7)	275 (67.7)	0.800
**BMI, Kg/m^2^**	26.6 ± 5.0	27.2 ± 5.3	0.190
**Neck circumference, cm**	39.0 ± 3.6	39.5 ± 3.2	0.137
**STOP-Bang score**			*0.035*
**Low (0–2)**	35 (19.8)	49 (12.1)	
**Intermediate (3–4)**	90 (50.8)	211 (52.0)	
**High (5–8)**	52 (29.4)	146 (36.0)	
**ASA**			0.767
**2**	84 (47.5)	184 (45.3)	
**3**	86 (48.6)	209 (51.5)	
**4**	7 (4.0)	13 (3.2)	
**Revised Cardiac Risk Index**			*0.004*
**1**	81 (45.8)	125 (30.8)	
**2**	65 (36.7)	200 (49.3)	
**3**	23 (13)	66 (16.3)	
**4/5**	8 (4.5)	15 (3.7)	
**Type of Surgery**			*<0.001*
**Intraperitoneal**	39 (22.0)	161 (39.7)	
**Vascular**	38 (21.5)	63 (15.5)	
**Orthopedic**	67 (37.9)	95 (23.4)	
**Other**	33 (18.6)	87 (21.4)	
**Type of anesthesia**			0.211
**GA**	120 (67.8)	302 (74.4)	
**Combined**	18 (10.2)	38 (9.4)	
**Neuraxial**	39 (22)	66 (16.3)	
**Duration of surgery**	2.8 ± 1.6	3.2 ± 2.2	*0.007*
**Preop AHI, events/hour**	14.4 ± 12.1	19.5 ± 14.4	*<0.001*
**Preop ODI, events/hour**	14.1 ± 11.4	19.0 ± 12.9	*<0.001*
**Postop ODI, events/hour**	15.4 ± 12.2	2.9 ± 3.1	*<0.001*
**Preop Average SpO_2_, %**	94.3 ± 2.2	93.9 ± 2.6	0.058
**Preop Lowest SpO_2_, %**	75.4 ± 12.6	75.0 ± 10.9	0.712
**Preop CT90, minutes**	31.1 ± 57.0	42.3 ± 66.2	*0.038*
**Preop CT80, minutes**	3.6 ± 8.7	7.1 ± 22.6	*0.007*

Independent sample *t*-tests used to compare the numerical values, and Chi-square tests used to compare categorical values. Responders to oxygen therapy were defined as individuals with a ≥50% reduction in postoperative oxygen desaturation index (ODI) versus preoperative ODI. Non-responders were defined as individuals with a <50% reduction in ODI. CT: cumulative time; SpO_2_: oxyhemoglobin; ODI: oxygen desaturation index; AHI: apnea hypopnea-index; ICU: intensive care unit; BMI: body mass index; OSA: obstructive sleep apnea; ASA: American Society of Anesthesiologists.

## Data Availability

No application.

## Supplementary Materials

The following supporting information can be downloaded at: https://www.mdpi.com/article/10.3390/jpm12101543/s1, Table S1: Characteristics of patients requiring oxygen therapy based on OSA severity.

## Group Information

The Postoperative Vascular Complications in Unrecognized Obstructive Sleep Apnea (POSA) Study Investigators: **Steering Committee:** Frances Chung (chair); Matthew Chan, Chew-Yin Wang, Edwin Seet. **Investigators: Canada:** *Scarborough Health Network—Central Campus:* Frances Chung, MBBS, Stanley Tam, MD, Sohail Iqbal, BSc; **Hong Kong:** *Prince of Wales Hospital:* Matthew Chan, MBBS, PhD, Gordon Choi, MBBS, David Hui, MD, Tony Gin, MD, Matthew Tsang, BSc, Beaker Fung, BSc, Angela Miu, BSc, Alex Lee, MSc; *Tuen Mun Hospital:* Benny Cheng, MBBS, Carmen Lam, MBBS, Sharon Tsang, MBChB, PhD, Chuen Ho Cheung, MBChB, Hoi Lam Pang, MBBS; **Malaysia:** *University Malaya Medical Centre:* Chew Yin Wang, MBChB, Hou Yee Lai, MBBS, Carolyn C.W. Yim, MBBS, Alvin S.B. Tan, MBBS, Ching Yen Chong, BA, Jason H. Kueh, BSc, Xue Lin Chan, MBChB; *Hospital Kuala Lumpur:* Eleanor F.F. Chew, MBBS, Su Yin Loo, MBBS, Simon M.T. Hui, MBBS; **New Zealand:** *Middlemore Hospital:* Joyce Tai, MBChB, Stuart Walker, MBBS, Sue Olliff, BSc; *Auckland City Hospital:* Ivan Bergman, MBBS, Nicola Broadbent, MBBS, Maartje Tulp, MBBS, Timothy Short, MD, Davina McAllister, BSc; **Singapore:** *Khoo Teck Puat Hospital:* Edwin Seet, MBBS, Pei Fen Teoh, MBBS, Audris Chia, BSc.

## Abbreviations

CI, confidence interval; CT90%, cumulative time of oxygen saturation <90%; CT80%, cumulative time of oxygen saturation <80%; ODI, oxygen desaturation index; OSA, obstructive sleep apnea; OR, odds ratio; SpO_2_, oxyhemoglobin saturation.
