# Supplementary material for: Characteristics of Patients with Unrecognized Sleep Apnea Requiring Postoperative Oxygen Therapy
Source: J Pers Med. 2022 Sep 20;12(10):1543. doi: 10.3390/jpm12101543 (PMC9605207; doi:10.3390/jpm12101543)
Supplement: Supplementary file 1 [file jpm-12-01543-s001.zip › jpm-1876496-supplementary.pdf]

Supplementary Table S1. Characteristics of patients requiring oxygen therapy based on OSA severity.

	Mild OSA	Moderate OSA	Severe OSA	P
**Number of patients**	314	173	96	-
**Gender, male**	197 (62.7)	115 (66.5)	81 (84.4)	<0.001
**Hypertension**	253 (80.6)	157 (90.8)	91 (94.8)	0.001
**STOP-Bang**	3.6 ± 1.2	4.1 ± 1.3	4.9 ± 1.14	<0.001
**Duration of oxygen therapy, hour**	12 ± 4.9	13.1 ± 6.4	12.8 ± 5.8	0.195
**Types of devices**				0.227
**Nasal cannula**	215 (68.5)	114 (65.9)	60 (62.5)	-
**Simple facemask**	79 (25.2)	38 (22.0)	25 (26.0)	-
**Non-invasive ventilation devices**	19 (6.1)	19 (11.0)	11 (11.5)	-
**Non-rebreathing mask**	1 (0.32)	2 (1.2)	0	-

Abbreviation: OSA, obstructive sleep apnea. Non-invasive ventilation devices include continuous positive airway pressure (CPAP), bilevel positive airway pressure (BPAP) ventilation. Post hoc analysis were conducted to examine the severity of AHI.
